# Assessment of changes in the municipal waste accumulation in Poland

**DOI:** 10.1007/s11356-020-08943-6

**Published:** 2020-04-30

**Authors:** Grzegorz Przydatek

**Affiliations:** Engineering Institute, State University of Applied Sciences in Nowy Sącz, Zamenhofa 1a street, 33-300 Nowy Sacz, Poland

**Keywords:** Municipal solid waste, Waste management, Mixed waste, Waste accumulation rate, Landfill, Gross domestic product

## Abstract

The aim of the work was to assess the effectiveness of municipal waste management in Poland over the period 2012–2017, considering the accumulation of waste collected selectively and non-selectively per capita and the changes resulting from the implementation of Directives 1999/31/EC and 2008/98/EC in Polish legislation. Within 6 years, noticeable changes in the country initiated by the EU and national legislation indicated an increase in the amount of waste to be recovered. However, the achieved efficiency of limiting the deposited waste at the level of 20% was moderate, despite the existence of infrastructure allowing for increased recovery. The analysis of the efficiency of waste management showed a certain convergence of the increase in the amount of generated waste and gross domestic product (GDP). On the other hand, the increase in accumulated organic waste per capita in all three dimensions of time was the most significant and exceeded 20%.

## Introduction

Currently, municipal solid waste (MSW) is an inevitable effect of society’s existence and the functioning of economic entities. For a society to live in an ecologically sustainable country, it is necessary to introduce waste management methods that consider waste recovery, environmentally safe disposal, and above all, minimisation of the quantity and effect of waste on the environment (Tsoulfas and Pappis 2006).

The public is obliged to adapt to the legal conditions that regulate these methods. From mid-2013 in Poland, in accordance with the implementation of Directives 1999/31/EC and 2008/98/EC, the generation of MSW must be prevented as much as possible, and waste recovery must be increased to 50% by 2020 through segregation at the source (Boas-Berg et al. 2018).

One of the factors preventing the mentioned waste production in both Poland and the European Union (EU) member states is the application of the waste hierarchy (Pomberger et al. 2017). This includes recycling, which limits the effect of waste on the environment, reduces the consumption of natural resources, and decreases costs (Eriksson et al. 2005). Waste management should be dominated by rational solutions, which should be consistent with the principles of environmental protection and material management (Przydatek 2012).

Waste accumulation rates play a key role in waste management and have been used by many researchers (Burnley 2007; Dahlén et al. 2007; Liikanen et al. 2016; Talalaj and Walery, 2015; Przydatek et al. 2017, 2018). According to Miliute-Plepiene and Plepys (2015), the number of studies considering waste accumulation rates is increasing, which may result from the need to identify the factors causing the mass increase of generated waste. The aim of the work was to assess the effectiveness of municipal waste management in Poland in 2012–2017, considering nine indicators of waste accumulation per capita within monthly and annual dimensions.

## Method and materials

The paper presents issues related to the effectiveness of waste accumulation in the context of changes in municipal waste management in Poland. In the first step, the literature was reviewed. Next, for quality and quantity analyses, data were selected from Statistics Poland (SP) for the period from 2012 to 2017. Based on these data, the general mass of municipal waste was divided into non-selectively and selectively collected waste, by the number of inhabitants and landfills, and by the value of the gross domestic product (GDP). Among the selectively collected waste, six categories of the most commonly collected municipal waste were included: paper and cardboard, glass, plastics, metals, used electrical and electronic equipment, and biodegradable waste. The paper addresses Directive 2008/98/EC, which requires selective waste collection, which should include at least paper, metal, plastic, and glass waste. The qualitative-quantitative analysis covered the annual sums of municipal waste collected in Poland in 2012–2017. On this basis, nine indicators of mass accumulation of waste on an annual, monthly, and daily bases were determined, which included the GDP, non-selectively collected waste, selectively collected waste, and the total waste and was divided into six separately collected waste categories. In addition, descriptive statistics covering the minimum, maximum, and average values were used in the work.

### Description of Poland

Poland lies in the north-eastern hemisphere between 49°00′N and 54°50′N latitude and between 14°07′E and 24°08′E longitude. It is the 69th in the world in terms of area with 316.6 thousand km^2^. In terms of population, it is the 36th in the world and the 9th in Europe, and the largest city (the capital) is Warsaw. The state, located in Central Europe, borders on Belarus, the Czech Republic, Lithuania, Germany, Russia, Slovakia, and Ukraine. The length of Poland’s border is 3511 km, including 440 km on the sea border (the Baltic Sea coastline). Poland in administratively divided into three levels, namely, 2500 communes, 380 counties, and 16 voivodeships. The Masovian Voivodeship in central Poland covers the largest area of 35,500 km^2^, while Opolskie Voivodeship has the smallest area in the southwestern part of the country, which is smaller by 26,100 km^2^. In the majority of the country, lowland areas cover the eastern part of the Central European Lowland with an average height of 173 m above sea level. The natural environment of Poland is extremely varied. The Baltic Sea is in the northern part, whereas lake districts and vast lowlands comprise the southern part. In the south, uplands and two mountain ranges dominate the area. In terms of GDP, Poland has the 6th ranked economy in the EU and the 25th in the world.

### Municipal waste management

At the national level, the National Waste Management Plan (NWMP) 2022 defines the main solutions in waste management (NWMP, 2016). This plan sets out the assumptions for voivodeship-level waste management plans. These plans set out municipal waste management regions and a list of regional municipal waste treatment facilities (RMWTFs) as well as installations intended for substitute service to these regions in the event of a breakdown. Local government units may specify assumptions regarding the scope of the projects and their location, with adaptation to the applicable plan at the voivodeship level (16 voivodeships exist). Municipal self-governments have introduced systemic changes in waste management. Since 2013, they are obliged to submit annual reports on the implementation of tasks in the field of municipal waste management. In the period preceding systemic changes, such reporting was prepared every 2 years on the basis of the communal level plans in force at the time.

In accordance with the hierarchy of waste management, the priority is to prevent waste or increase recovery and to reduce the number of landfills (Przydatek 2012). The fundamental changes in the waste management system have resulted from the implementation of Directives 1999/31/EC and 2008/98/EC into Polish legislation by imposing an obligation on the community to organise a municipal waste collection system for property on which residents live, with the possibility of extending this system to other properties in which municipal waste is generated, considering the specific cost. Somplak et al. (2014) showed that the storage and incineration of waste primarily determine the amount of the fee in the MSW management systems.

An important factor in changes in the waste management system at the national level was the introduction of changes through the act on 1 July 2011 amending the act on maintaining cleanliness and order in communes and certain other acts, which entered into force on 1 January 2012. On this basis, all residents were covered by the selective collection of waste. At the same time, measures were taken to limit the storage of raw material waste, including biodegradable waste, by up to a significant level of 35% by 2020. Without further development of the selective collection and processing of waste, including organic waste, and construction of installations for processing mixed municipal waste, it would not be possible to limit their storage (Przydatek 2012). At the end of 2014, there were 769 waste disposal installations in Poland: 391 landfills, 127 installations of mechanical and biological waste treatment (MBP), 97 installations for processing selectively collected green waste and other bio-waste. The only thermal waste treatment plant, which operates in Warsaw, has a capacity of 60,000 Mg per year (NWMP 2016). In major cities, incinerators in Poznań, Szczecin, Kraków, Białystok, and Bydgoszcz remain under construction (Górnicki 2014). Most of the 13 MBP installations are operating in central and southern Poland (Masovian and Lesser Voivodeships), and there are 10 installations for processing of selectively collected green waste and other bio-waste in northern and southern Poland (Kuyavian-Pomeranian, Pomeranian, and Lesser Voivodeships).

In Poland, waste disposal can be carried out in a non-replaceable system, which is associated with emptying containers near their place of manufacture and an exchange system including the substitution of containers or empty containers at the place of collection using export rolling stock (Przydatek 2012). Generally, waste is broken down into selectively and non-selectively collected waste. The selective categories include paper and cardboard, glass, plastics, metals, and organic material. Then, they take them into RMWTFs or installations intended for substitute service for the region. Municipal waste is collected in containers and is sporadically collected in bags on properties. In other EU countries (Gallardo et al. 2010), the collection of waste considers the possibility of joint collection and disposal of paper and board waste as well as metals and plastics. In Spain, the mixed waste is differently placed in containers placed at the street kerb.

The costs of the municipal waste management system include collection, transport, recovery, and disposal of municipal waste. In addition, the costs include the creation and maintenance of separate collection points for municipal waste and system administration. The fees for municipal waste management are adopted by municipal councils, considering the number of residents living in the commune and the amount of municipal waste produced in the commune (Przydatek 2012). Unit rates for waste disposal and disposal are diversified in terms of price. Higher rates tend to dominate in the case of waste collected non-selectively. In other countries, there is a tax for the collection and management of waste, which is calculated based on the weight/volume of the waste (Abbott et al. 2011).

## Results and discussion

The type and area of the generated waste are also influenced by the type of area in which it is produced, the population density, the type of building, the presence of public facilities, and the presence of retail outlets and small industry or services (Przydatek 2012). This confirms that the recognition of the efficiency of waste management depends on many factors. However, the basis for this diagnosis both on a global and national scale is the amount of waste generated in a given time perspective.

**Fig. 1:**
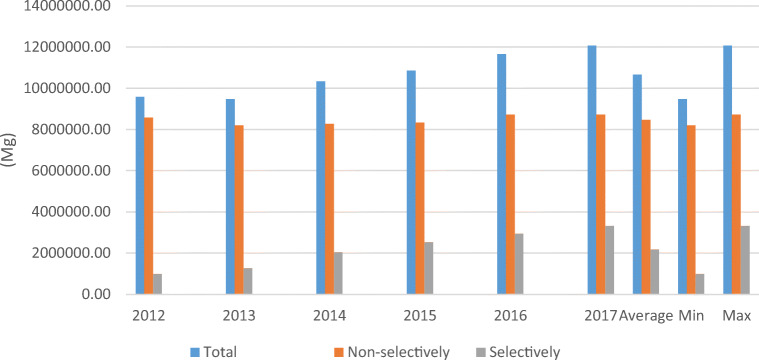
Amount of collected waste in Poland (SP, 2012–2017)

Figure 1 presents data on the total amount of collected municipal waste in 2012–2017 divided into selectively and non-selectively collected waste. In general, during the analysed period, the amount of collected waste increased by 2,477,800 Mg (which is 26%), including the highest amount at 2,324,000 Mg for selectively collected waste. The demonstrated trend of the increase in the amount of waste in Poland is consistent with the global trend, which is characterised by a rapid increase in quantity (Hoornweg and Bhada-Tata 2012).

Another researcher (Przydatek 2015) found that the increase in the amount of generated waste is associated with the increase in consumption by residents. A favourable trend of waste recovery occurred when, in the same period, a noticeable decrease occurred in the number of residents in the country, by as many as 99,700 (SP 2012–2017). Such an increase in the amount of generated waste also occurred in Japan (Hotta and Aoki-Suzuki 2014). Alternatively, the increase by 153,800 Mg (1.79%) in the amount of waste collected non-selectively was unfavourable. Hannan et al. (2015) reported that an increase in the amount of MSW may be associated with fast-paced urbanisation. In general, the increase in the amount of waste was generated in line with the increase in GDP per capita in 2012–2016 by EUR 1.427 (Table 1). Górnicki (2014) noticed a declining trend of GDP in Poland in 2004–2008. Such changes may indicate that, within the framework of the national pro-ecological policy, actions are taken to consolidate such trends to subject the largest amount of waste generated to technological processes that will allow compliance with the EU environmental policy criteria.

**Table 1:** Gross domestic product in Poland in 2012–2017

Gross domestic product per capita					
2012	2013	2014	2015	2016	2017*
(PLN)					
42,285	43,034	44,705	46,814	48,432	
(EUR)					
9815	9989	10,377	10,866	11,242	

Average rate EUR/PLN 43082

*Lack of data

**Fig. 2:**
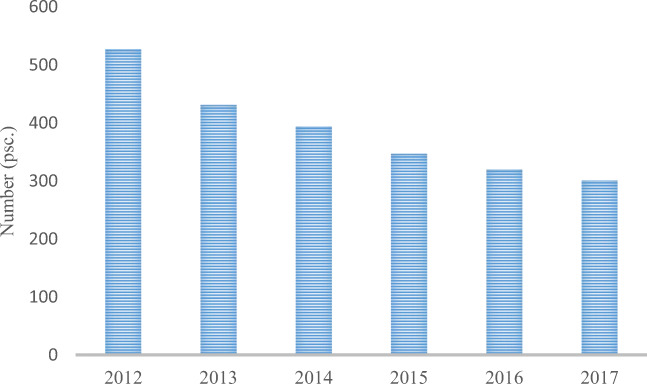
Amount of used landfills in Poland in 2012–2017 (SP, 2012–2017)

The gradual changes in waste management are indicated by the lowest amount of collected waste, which usually occurred at the beginning of the research period in 2012 (selectively collected) and 2013 (overall and non-selectively collected). The highest amount was observed in the 2017 (the last considered year). In domestic conditions, the increase in the amount of waste after 2013 was shown by Przydatek (2019) in his research. This initial period did not include grounded changes in waste management, the moderate effect of which was noticeable in 2017. The short implementation period of Directives 1999/31/EC and 2008/98/EC since mid-2013 has shown unsatisfactory efficiency of waste management due to the high average volume of 8,470,400 Mg (79.5%) of non-selectively collected waste. Bing et al. (2016) pointed out that, in the new EU countries, the storage of waste dominates, which is above the average of 40% of this form of waste management in the EU. In the analysed period, a significant decrease occurred in the number of active landfills (226) in the country (which constitutes 43%; Fig. 2). Despite the indicated changes, the amount of selectively collected waste in relation to the non-selectively collected waste was unfortunately lower by 6,280,600 Mg with the use of 127 installations of regional MBP waste treatment facilities and a processing capacity of 13,510,000 Mg per year, which testifies to the existence of a significant surplus of the installation’s capacity (NWMP 2016).

**Fig. 3:**
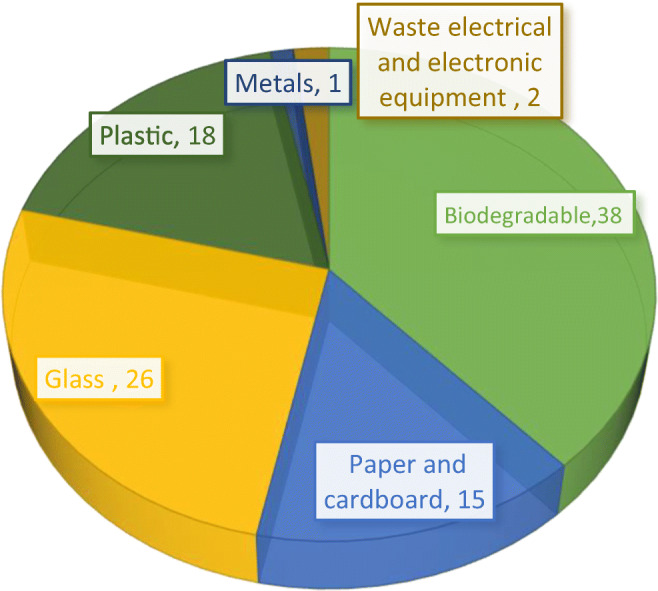
Contribution of fractions selectively collected waste

The amount of waste selectively collected and the proportion divided into the six categories are presented in Table 2. The highest average amount of selectively collected waste at 315,700 Mg (38%) was biodegradable waste (Fig. 3). At the national level, Przydatek et al. (2017) also confirmed a significant percentage of organic waste. This result was associated with the largest increase in the weight of selectively collected waste, which exceeded 2,000,000 Mg in relation to the base year. In 2017, the largest amount of organic waste was collected, which amounted to 895,300 Mg (26%) and simultaneously recorded the highest waste accumulation per capita. The achieved level of organic waste recovery should be considered favourable due to the requirements imposed on limiting organic waste storage to 35% in 2020 (NWMP 2016). A noticeable trend occurred in the increase in the amount of collected biodegradable waste, which amounted to 693,700 Mg against the processing capacity of 97 installations for selective processing collected green waste and other bio-waste amounting to 873,231,300 Mg per year. This indicates the existence of a provision that allows an increase in the level of recovery of the indicated waste, and thus the possibility of reducing organic waste storage.

**Table 2:** Amount and contribution fractions of segregated waste in Poland in 2012–2017

Segregated waste							
Year	Unit	Biodegradable	Paper and cardboard	Glass	Plastics	Metals	WEEE
2012	(Mg)	201,629.40	186,645.70	275,590.40	176,391.80	14,399.20	21,998.10
	(%)	6	14	12	11	13	12
2013		311,787.20	196,720.70	316,166.80	219,617.90	18,151.00	27,138.90
		9	15	14	14	16	15
2014		583,670.00	240,476.20	411,097.00	314,182.50	20,382.70	25,698.70
		17	18	18	19	18	15
2015		657,047.50	243,155.50	424,103.30	303,224.70	19,214.80	29,224.40
		19	18	18	19	17	17
2016		822,864.00	254,078.10	447,270.30	304,208.40	24,251.00	30,429.20
		24	19	19	19	22	17
2017		895,394.80	230,561.30	462,997.40	295,308.90	14,972.60	41,757.20
		26	17	20	18	13	24
Average	(Mg)	3472,392.90	1351,637.50	2337,225.20	1612,934.20	111,371.30	176,246.50
Min.	(Mg)	315,678.829	122,883.677	212,482.308	146,637.808	10,132.532	16,029.346
Max.	(Mg)	201,629.40	186,645.70	275,590.40	176,391.80	14,399.200	21,998.10

In addition, the highest amount of recovered glass waste was recorded in 2017, which was almost half that of the average organic waste category. The average weight of glass on a national scale did not exceed 3,000,000 Mg and was higher by 65,800 Mg and 89,600 Mg, respectively, in relation to the average amounts of collected plastic waste and paper and cardboard waste. Such changeability of waste recovery may result from the purchase price of waste intended for recycling, which determines the competitiveness of recycling initiatives (Bing et al. 2016). Between the two categories, there was a slight 3% difference in the content, indicating the dominance of plastics (Fig. 3). Nevertheless, the amount of paper and board waste in 2012–2016 increased by 67,400 Mg. According to Ishchenko et al. (2017), such growth may have been caused by the increase in the level of paper waste recycling, which is widely used in new products. After 2016, the weight of collected paper and cardboard waste decreased by 23,500 Mg with a decrease in content by 2%. The amount of plastic waste fluctuated significantly. In 2012–2014, an increase by 137,700 Mg occurred, whereas a decrease by 7900 Mg occurred after 2014.

The amount of metal waste in 2012–2014 increased significantly by over 6000 Mg. The largest share of 22% was found in 2016 at the highest value of 24,200 Mg. In 2017, a decrease of 9300 Mg occurred. According to Bing et al. (2016), reported decreases in waste recovery may be dependent on external factors, such as rising oil prices, cost dynamics, and various interests of homeowners and municipalities. The average amount of metal waste was the lowest and did not exceed 11,000 Mg. The lowest amount of each of the mentioned waste categories occurred simultaneously in 2012, and the lowest share of 1% of the six waste categories was attributable to metal waste (Fig. 3).

A valuable tool in recognising the efficiency of waste management at the national level was to consider different indicators of waste accumulation per capita. Table 3 presents indicators of waste accumulation both selectively and non-selectively per capita. The indicator of total waste accumulation, classified as one of the relevant indicators for a given country, has confirmed an increase of 65.12 kg per capita per year within 6 years. The best result of 313.75 kg per capita occurred in the last analysed year with an average value of 277,139 kg. However, the result of waste accumulation per capita in the country in 2016 in comparison with the values achieved in the EU was lower with the exception of less than 200 kg per capita per year in Romania (Eurostat 2016). The amount of generated waste had a significant influence on the value of the indicator. In 2012–2015, the number of inhabitants decreased by 0.25% with a simultaneous increase in GDP by 14.5%. Two indicators of selective waste accumulation confirmed the general increase, which reached higher values in relation to the accumulation of mixed waste by 55.96 kg per capita per year and 4.66 kg per month. On a daily basis, this difference was 0.15 kg.

**Table 3:** Waste accumulation per capita in Poland in 2012–2017

	Accumulation rate								
Year	Total waste			Mixed waste			Segregated waste		
	(kg year^−1^)	(kg month ^−1^)	(kg day^−1^)						
2012	248.64	20.72	0.68	222.55	18.55	0.61	26.09	2.17	0.07
2013	246.10	20.51	0.67	212.98	17.75	0.58	33.12	2.76	0.09
2014	268.47	22.37	0.74	215.22	17.93	0.59	53.26	4.44	0.15
2015	282.63	23.55	0.77	216.61	18.05	0.59	66.01	5.50	0.18
2016	303.24	25.27	0.83	226.68	18.89	0.62	76.56	6.38	0.21
2017	313.75	26.15	0.86	227.13	18.93	0.62	86.63	7.22	0.24
Average	277.139	23.095	0.759	220.194	18.350	0.603	56.945	4.745	0.156
Min.	246.10	20.51	0.67	212.98	17.75	0.58	26.09	2.17	0.07
Max.	313.75	26.15	0.86	227.13	18.93	0.62	86.63	7.22	0.24

Table 4 presents the results of selective waste accumulation per inhabitant of Poland in 2012–2017. On the basis of the six analysed accumulation rates, the largest increase of 18.06 kg was organic waste per capita annually, despite the general decline in the number of residents in the country. Some researchers (Matsumoto 2011; Manaf et al. 2009) have justified such an increase in waste accumulation based on the increase in the number of inhabitants. Other indicators of waste accumulation in monthly and daily terms were lower: 12- and 30-fold. The next highest value of the accumulation rate concerned glass waste, which amounted to 12.05 kg per capita in 2017. The accumulation of plastic waste was characterised by an advantage over the average value of the glass waste accumulation rate. The average value of waste of electrical and electronic equipment accumulation rate amounted to 0.764 kg, and metal waste was 0.403 kg per capita per year, which is considered low. The latter rate reached the lowest average values broken down by month at 0.040 kg and daily at 0.001 kg. Such results confirm that the collection of this waste was concerning a smaller number of inhabitants, which could have been due to the smaller ecological awareness of waste producers (Ekere et al. 2009).

**Table 4:** Selectively waste accumulation rate per capita in Poland in 2012–2017

	Accumulation rate per capita																	
Year	Biodegradable waste			Paper and cardboard waste			Glass waste			Plastics waste			Metals waste			Waste electrical and electronic equipment		
	(kg year^−1^)	(kg month ^−1^)	(kg day^−1^)															
2012	5.23	0.44	0.014	4.84	0.40	0.013	7.15	0.60	0.020	4.58	0.38	0.013	0.37	0.03	0.001	0.57	0.05	0.002
2013	8.10	0.67	0.022	5.11	0.43	0.014	8.21	0.68	0.023	5.71	0.48	0.016	0.47	0.04	0.001	0.70	0.06	0.002
2014	15.17	1.26	0.042	6.25	0.52	0.017	10.68	0.89	0.029	8.17	0.68	0.022	0.53	0.04	0.001	0.67	0.06	0.002
2015	17.09	1.42	0.047	6.33	0.53	0.017	11.03	0.92	0.030	7.89	0.66	0.022	0.50	0.04	0.001	0.76	0.06	0.002
2016	21.41	1.78	0.059	6.61	0.55	0.018	11.64	0.97	0.032	7.92	0.66	0.022	0,63	0.05	0.002	0.79	0.07	0.002
2017	23.30	1.94	0.064	6.00	0.50	0.016	12.05	1.00	0.033	7.68	0.64	0.021	0.39	0.03	0.001	1.09	0.09	0.003
Average	15.050	1.254	0.041	5.857	0.488	0.016	10.128	0.844	0.028	6.989	0.582	0.019	0.483	0.040	0.001	0.764	0.064	0.002
Min	5.23	0.44	0.014	4.84	0.40	0.013	7.15	0.60	0.020	4.58	0.38	0.013	0.37	0.03	0.001	0.57	0.05	0.002
Max	23.30	1.94	0.064	6.61	0.55	0.018	12.05	1.00	0.033	8.17	0.68	0.022	0.63	0.05	0.002	1.09	0.09	0.003

## Conclusions

Based on the analysis of the effectiveness of municipal waste accumulation in Poland, considering the number of inhabitants, the amount of selectively and non-selectively collected waste, and indicators of waste accumulation, the following conclusions were formulated:

- In 6 years, the increase in the amount of waste generated is noticeable with a decrease in the number of inhabitants by almost 100,000 and an increase in the number of landfills by over 200.
- The GDP growth of 14.5% was accompanied by an increase in the amount of collected waste by 25%, which can be considered a significant factor.
- Waste management efficiency remains unsatisfactory due to the high proportion of up to 80% mixed waste despite the processing capacity for waste recovery installations, exceeding the demonstrated demand.
- Organic waste was the highest average category of the six separately collected types of waste, exceeding 500,000 Mg.
- The average value of organic waste accumulation per capita was the highest at 15.050 kg per capita per year. The lowest value was attributable to metal waste, broken down by month and day.
- The amounts of other collected waste categories were characterised by variable content that could be caused by the change in the preferences of waste producers.
